# PD-L1 Expression in Medullary Thyroid Carcinoma and Its Association with Clinicopathological Findings

**DOI:** 10.5146/tjpath.2021.01558

**Published:** 2022-05-19

**Authors:** Yasemin Kemal, Sultan Caliskan, Seda Gun, Mehmet Kefeli

**Affiliations:** Department of Medical Oncology, Istinye University, Faculty of Medicine, İstanbul, Turkey; Department of Pathology, Ondokuz Mayıs University, Faculty of Medicine, Samsun, Turkey

**Keywords:** Medullary thyroid carcinoma, Immunotherapy, Programmed death-ligand 1 (PD-L1), Clinicopathological characteristics

## Abstract

*
Objective:* Medullary thyroid carcinoma (MTC) is a rare tumor originating from parafollicular C cells. It has more aggressive biologic behavior than differentiated thyroid carcinomas, and it is insensitive to treatment with radioactive iodine. Vandetanib and cabozantinib are the newly approved tyrosine kinase inhibitors in advanced stages, but novel effective systemic therapeutics could be crucial and needed for the clinical management of these patients. We aimed to evaluate the Programmed death-ligand 1 (PD-L1) expression, which is a novel immunotherapy target, in our MTC cohort, and determine whether it has an association with clinical and pathological features.

*
Material and Method:* This retrospective study involved 41 cases of MTC with a median follow-up of 54 months. PD-L1 monoclonal antibody (SP263 clone) was investigated immunohistochemically. Complete and/or partial membranous staining pattern in more than 1% of tumor cells was considered positive. The correlations of PD-L1 expression with clinicopathologic and prognostic features were analyzed.

*
Results:* PD-L1 positivity was detected in 5 (12.2%) of 41 tumors. The extent of PD-L1 staining was low (<5%) for all tumors. There was no clinicopathologic and prognostic relevance regarding PD-L1 expression in our MTC patients.

*
Conclusion:* Although PD-L1 expression could be a potential biomarker to predict the prognosis of various cancers and response to checkpoint inhibitors, we did not find any significant correlation between PD-L1 expression and clinicopathologic features in our cases. Studies with larger patient numbers are still required to perform a more comprehensive analysis.

## INTRODUCTION

Medullary thyroid cancer (MTC) is a rare neuroendocrine tumor originating from calcitonin-producing parafollicular C cells of the thyroid gland. It has an aggressive clinical course and a worse prognosis than differentiated thyroid carcinomas, and the 10-year survival rate is reported as 45-85% (1–5). The prognosis is usually associated with the clinicopathological findings (i.e., age, gender, the presence of local tumor invasion, lymph node metastases, and distant metastases) and the mutant codon region of the *RET* gene (i.e., M918T) (3–7). The current initial treatment approach of MTC is total thyroidectomy and central compartment lymph node dissection. Systemic treatment is considered for patients with significant tumor burden or progressive or unresectable metastatic disease. Because cytotoxic chemotherapeutics, selective RET inhibitors, and multi-kinase inhibitors have low/modest response rates and significant toxicities, and new and effective treatment agents are needed for advanced progressive MTC (3).

Immunotherapy with checkpoint inhibitors targeting Programmed death receptor-1 (PD-1) and Programmed death-ligand 1 (PD-L1) have been used effectively for the treatment of various tumors as a promising alternative for cancer management and found to be associated with the prognosis of the patients (7–12). The relationship between PD-L1 and thyroid carcinoma was also investigated; however, nearly all these studies included differentiated thyroid carcinomas (13–20). The knowledge about the PD-L1 expression in MTCs is limited; it has been evaluated only in a few studies to date. In this study, we aimed to investigate PD-L1 expression in MTC patients treated at our university hospital. We also investigated the association between PD-L1 positivity and the clinicopathological characteristics of the patients’.

## MATERIALS and METHODS

A total of 41 MTC patients diagnosed between 2009 and 2018 were included in the study. Institutional REB (research ethics board) approval was obtained. The diagnosis of MTC is proved based on cytomorphologic features accompanying diffuse immunoreactivity for monoclonal CEA and calcitonin. Four µm sections were taken from paraffin blocks representing selected patients, and sections were investigated with PD-L1 monoclonal antibody (SP263 clone, Ventana Medical Systems, Inc., AZ) using a Benchmark XT automated staining device (Roche, Ventana Medical Systems). Because MTCs usually have very few tumor-associated inflammatory cells, PD-L1 expression was determined by using the tumor proportion score rather than the combined positive score. In all sections, PD-L1 expression in tumor cells was evaluated. PD-L1-expressing tumor cells were scored as a percentage of total tumor cells. Complete and/or partial membranous staining pattern in tumor cells was considered positive. Positive staining in more than 1% of tumor cells was determined as the threshold value for positive staining. The patients with positive PD-L1 staining were separated into low (1-5%) and high (>5%) PD-L1 positive groups. The PD-L1 stained slides were evaluated by a pathologist without knowledge of the clinicopathologic features. Normal human term placental tissue was used as the control according to the manufacturer’s suggestion because it contains positive and negative staining elements for the PD-L1 protein.

PD-L1 staining was compared with the following parameters: age, gender, tumor diameter and localization, pT and pN status, multifocality, bilaterality, the presence of capsule, capsule invasion, surgical margin status, vascular invasion, lymphatic invasion, perineural invasion, extrathyroidal extension, lymph node involvement, C cell hyperplasia, the presence of chronic lymphocytic thyroiditis, and recurrence. Histopathologically or cytologically confirmed relapses of the patients with normal radiological findings and normal-decreased serum calcitonin levels after initial surgery were considered recurrence. Recurrence was found in nine out of 41 patients, and all of them were detected at the regional lymph nodes.

SPSS, version 20 was used for statistical analysis. Fisher’s exact test were used to compare the PD-L1 immunohistochemical results with parameters such as age, gender, tumor size, multifocality, surgical margin status, pT stage, pN stage, and recurrence. In the statistical analysis, NA (not-available) data were considered missing data, and statistical analysis was performed by excluding these data. A p-value of less than 0.05 was considered significant.

## RESULTS

The age range at diagnosis of the patients was between 12 and 94 years (mean age: 47.8; median age: 48) for the overall cohort. Twenty-four patients were female, and seventeen patients were male. The mean tumor diameter was 22.2 ±12.7 mm (range: 1-55 mm). Twenty-four tumors (58.5%) were unifocal. According to the TNM stage, twenty-three patients were pT1, sixteen patients were pT2, and two patients were pT3 status. Lymph node dissection was performed for 21 out of 41 patients (51.2%). Nine patients were pN0, six patients were pN1a, six patients were pN1b, and twenty patients were pNx. Recurrence was seen in nine of 41 patients, and all of them were detected at the regional lymph nodes. None of the 41 patients had distant metastases at diagnosis. All the patients were alive during the data collection phase.

**Figure 1:**
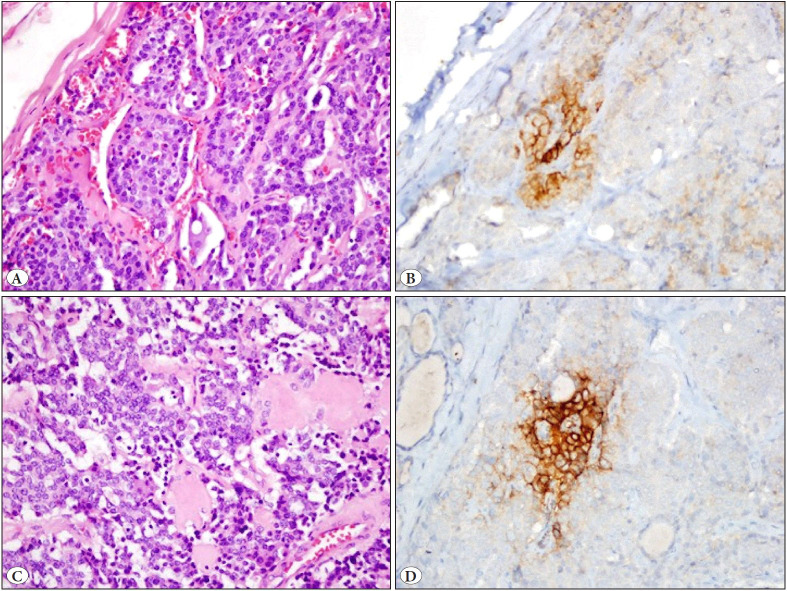
Focal (<5%) membranous PD-L1 expression in medullary thyroid carcinomas (**A,C:** H&E; x20&40; **B,D:** PD-L1; x20&40).

**Figure 2:**
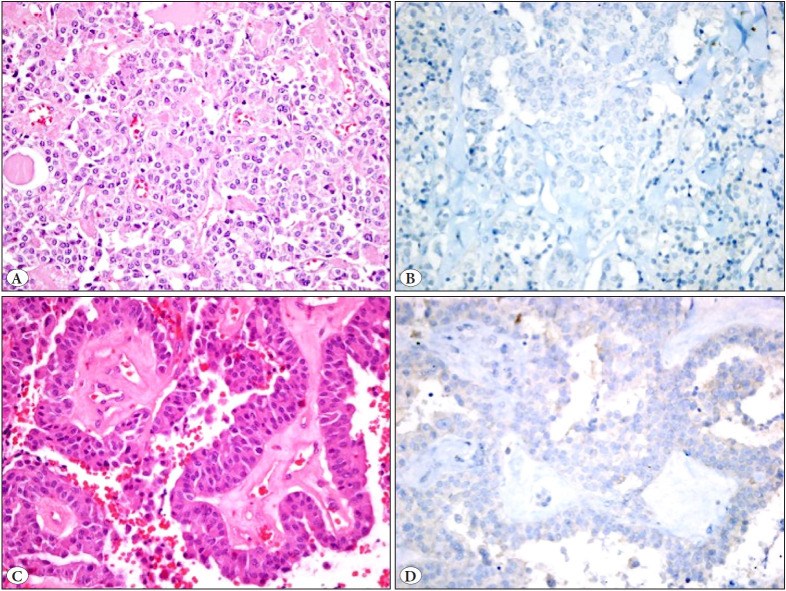
PD-L1 negative medullary thyroid carcinomas (**A,C:** H&E; x20&40; **B,D:** PD-L1; x20&40).

PD-L1 positivity of tumor cells was detected in 5 (12.2%) of 41 tumors (Figure 1A-D, Figure 2A-D). The extent of PD-L1 staining was low (1-5%) for all tumors. The age range at the time of diagnosis in PD-L1 positive patients was between 32 and 67 years (mean age: 47.8; median age: 35). One of the PD-L1 positive patients was male, and four were female. The tumor was located in the right lobe in three of the PD-L1 positive patients and in the left lobe in two patients. Multifocality, bilaterality, and separate tumor foci were observed in one patient. The tumor was encapsulated in two patients, and all tumors had capsule invasion. In all PD-L1 positive tumors, the surgical margin was intact, and no perineural invasion or extrathyroidal extension was observed. Three patients had C cell hyperplasia, and two patients had lymph node metastasis. One of five PD-L1 positive patients also had a chronic lymphocytic thyroiditis background. There was no correlation between PD-L1 negative and positive patients based on clinicopathological characteristics, including age, sex, tumor size, multifocality, surgical margin, pT stage, pN stage, initial lymph node metastasis, recurrence, and relation with chronic lymphocytic thyroiditis (p>0.05). Correlation between PD-L1 expression and clinicopathological characteristics are shown in Table 1 and Table 2. There was no clinicopathologic and prognostic relevance regarding PD-L1 expression in this cohort. The median follow-up time was 54 months (min. 15 months, max. 127 months).

**Table 1:** Clinicopathological features of 41 medullary thyroid carcinomas according to PD-L1 status.

**Variables**	**PD-L1 immunohistochemistry in tumor**		**Total**	p-value*
	**Negative, n (%)**	**Positive, n (%)**		
**Age (Year)**				
<50	19 (82.6)	4 (17.4)	23	0.363
≥50	17 (94.4)	1 (5.6)	18	
**Gender**				
Female	20 (83.3)	4 (16.7)	24	0.382
Male	16 (94.1)	1 (5.9)	17	
**Tumor size**				
≤2 cm	21 (91.3)	2 (8.7)	23	0.638
>2 cm	15 (83.3)	3 (16.7)	18	
**Multifocality**				
No	20 (83.3)	4 (16.7)	24	0.382
Yes	16 (94.1)	1 (5.9)	17	
**Surgical margin**				
Negative	30 (85.7)	5 (14.3)	35	1.000
Positive	6 (100.0)	0 (0.0)	6	
**pT stage**				
T1	21 (91.3)	2 (8.7)	23	0.638
T2-T3	15 (83.3)	3 (16.7)	18	
pN stage**				
N0	9 (100.0)	0 (0.0)	9	0.143
N1a	6 (100.0)	0 (0.0)	6	
N1b	4 (66.7)	2 (33.3)	6	
Initial LN metastasis**				
Yes	14 (87.5)	2 (12.5)	16	1.000
No	5 (100.0)	0 (0.0)	5	
**Recurrence**				
No	28 (87.5)	4 (12.5)	32	1.000
Yes	8 (88.9)	1 (11.1)	9	
**Chronic lymphocytic thyroiditis**				
Yes	7 (87.5)	1 (12.5)	8	1.000
No	29 (87.9)	4 (12.1)	33	

*Fisher’s Exact Test, p>0.05, **20 cases of unknown initial lymph node status were not included.

**Table 2:** Clinicopathological features of PD-L1 positive medullary thyroid carcinomas.

**Case No**	**PD-L1 in tumor (%)**	**Age**	**Gender**	**Size of the largest tumor (mm)**	**Multiplicity**	**pT stage**	**pN stage**	**Recurrence**	**Death**
**1**	1-5	32	Female	32	No	pT2	pN1b	No	Alive
**2**	1	35	Female	30	No	pT2	pNX	No	Alive
**3**	1-5	36	Female	17	Yes	pT1b	pN1b	Yes	Alive
**4**	1	67	Male	18	No	pT1b	pNX	No	Alive
**5**	1	32	Female	25	No	pT2	pNX	No	Alive

## DISCUSSION

The expression of PD-L1 in thyroid cancers originating from follicular cells has been investigated in many recent studies, and the rates of PD-L1 positivity vary between 6.1% and 82.5% in thyroid carcinomas originated from follicular cells (13). The investigator found that PD-L1 positivity was higher in anaplastic thyroid carcinoma than in papillary thyroid carcinoma and follicular thyroid carcinoma, and it is associated with a worse prognosis and aggressive tumor behavior (13). Some studies have indicated that it could be a potential target in the management of thyroid cancer with high expression of PD-L1 (13–20). In the recent meta-analytic study, Girolami et al. provided a systematic review of the published data relevant to follicular epithelial-derived thyroid carcinoma. They reviewed 445 manuscripts and included 15 of them that had sufficient data to perform a quantitative analysis. The study’s results indicated that PD-L1 expression was significantly associated with reduced disease-free survival, but no association was found with the overall survival. Besides, they found a significant association between PD-L1 expression in papillary thyroid carcinomas in terms of underlying chronic lymphocytic thyroiditis and BRAF V600E mutation status (21). Nevertheless, the knowledge of the relationship between PD-L1 and MTC is less than the tumors originating from follicular cells; only a few studies have evaluated PD-L1 positivity in MTC patients (22–24)).

Bongiovanni et al. first investigated the expression of PD-L1 in MTC, and PD-L1 expression was detected in only 1 of 16 MTC patients (6.2%) (22). In the second study, Bi et al. reported 21.8% positivity among 81 MTC patients (23). More recently, a large cohort study revealed that 29 of 201 patients (14.4%) showed positive staining by the PD-L1 antibody (24). In another study, Ingenwerth et al. reported that neither tumor cell nor lymphocytes/macrophages showed PD-L1 expression in 38 MTC patients in their cohort (25). In our study, the percentage of PD-L1 expression was 12.2%. The positive staining rate between these five studies varies between 0% and 21.8%, and this alteration may be due to differences in the evaluation method and antibodies used. In those studies, the 22C3 clone was used in two studies, and the SP263 clone was used in three studies, including the current study. In non-small cell lung carcinomas, reported PD-L1 expression for the clone of SP263 was higher than for the clone of 22C3 (26). Therefore, the use of different clones could affect the positivity of tumor cells in studies. Different case numbers and clinicopathological features of the patients (e.g., different numbers of included progressive metastatic or advanced stage patients in the cohorts) in studies may also cause staining rates to differ. A comprehensive summary of the published studies of the PD-L1 expression in medullary thyroid carcinoma is shown in Table 3.

**Table 3:** Comprehensive summary of the published studies of the PD-L1 expression in medullary thyroid carcinoma.

	**Current study** **(n:41)**		**Bongiovanni et al.** **(n:16)**			**Shi et al.** **(n:201)**		**Bi et al.** **(n: 87)**		**Ingenwerth et al.** **(n:38)**
	**PD-L1 IHC in tumor cells**		**PD-L1 IHC in tumor cells**			**PD-L1 IHC in tumor and immune cells**		**PD-L1 IHC in tumor cells**		**PD-L1 IHC in tumor and immune cells**
	Clone SP263, Ventana Medical Systems, Inc., AZ		Clone SP263, Ventana Medical Systems, Inc., AZ			Clone 22C3, Dako, Carpinteria, CA		Clone SP263, Ventana Medical Systems, Inc., AZ		Clone 22C3, Dako, Carpinteria, CA
**Variables**	**Negative** **(n:36)**	**Positive** **(n:5)**	**Negative** **(n:15)**		**Positive** **(n:1)**	**Negative** **(n:172)**	**Positive** **(n:29)**	**Negative** **(n:68)**	**Positive** **(n:19)**	**Negative** **(n:38)**
**Age at diagnosis (year)**										
<50	19	4	9	0		48 (12‐80)	49 (32-NA)	47.09 ± 12.59	50.16 ± 9.77	20
≥50	17	1	6	1						16*
**Sex**										
Female	20	4	10	1		84	11	35	8	21
Male	16	1	5	0		88	18	33	11	15*
**Tumor size**										
≤2 cm	21	2	5	1		105	10	2.35 ± 1.61	2.43 ± 1.44	NA
2.1-4 cm	13	3	6	0		50	18			
>4 cm	2	0	4	0		17	1			
**Multifocality**										
No	20	4	NA	NA		127	22	62	19	NA
Yes	16	1				45	7	6	0	
**Extrathyroidal extension**										
No	30	5	NA	NA		130	22	NA	NA	NA
Yes	0	0				42	7			
NA	6	0				0	0			
**Surgical margin**										
Negative	30	5	NA	NA		NA	NA	NA	NA	NA
Positive	6	0								
**pT stage**										
pT1	21	2	4	1		NA	NA	NA	NA	NA
pT2	13	3	3	0						
pT3	2	0	7	0						
pT4	0	0	1	0						
**pN stage**										
pN0	9	0	4	0		59	5	NA	NA	NA
pN1a	6	0	8	0		46	5			
pN1b	4	2		0		67	19			
pNx	17	3	3	1		0	0			
**Recurrence**										
No	28	4	NA	NA		NA	NA	NA	NA	NA
Yes	8	1				109**	34**			
**Chronic lymphocytic thyroiditis**										
Yes	7	1	NA	NA		NA	NA	NA	NA	NA
No	29	4								
**Status**										
Dead	0	0	7	0		8	4	8		NA
Alive	36	5	6	1		NA	NA	NA	NA	
NA	0	0	2	0						

**IHC:** Immunohistochemistry, **NA:** Not-available, **n: **Number of patients * Not-available for two patients. ** Structural and biochemical recurrence were evaluated separately.

Considering the relationship between PD-L1 positivity and the clinicopathological features of patients, different results have been reported in previous studies. In our cohort, there was no association between clinicopathologic features and PD-L1 expression. Bongiovanni et al. reported only one PD-L1 positive patient who had a microcarcinoma (0.8cm) and who was reported as alive at the last follow-up (22). Bi et al. reported that PD-L1 positivity was significantly correlated with distant metastases at surgery, and co-expression of PD-1 and PD-L1 is associated with a more advanced TNM stage (23). Similarly, in the study conducted by Shi et al., PD-L1 positive patients were associated with aggressive clinicopathological features such as larger tumor size, lymph node metastases, and advanced TNM staging (24). Although the rate of PD-L1 positivity in MTC patients varies in previous studies, some studies have emphasized that it can be a predictor for locally advanced disease and aggressive course.

PD-1/PD-L1 interaction plays a critical role in the immunosuppressive tumor microenvironment. This interaction inhibits T-cell activation and tumor lysis. It may explain the aggressive disease course and the higher stage. PD-L1 is thought to act as a predictive marker for anti-PD-1/PD-L1 immunotherapy, and there are several ongoing immunotherapy trials with clinical promise in thyroid cancers. According to a recent meta-analysis, PD-L1 positivity is correlated with poor prognosis in non-medullary thyroid cancer patients, and this result is consistent with many other human cancers (27).

There is no effective treatment option that prolongs overall survival in advanced stage MTC patients. Cytotoxic chemotherapy has low response rates and durations. Vandetanib and cabozantinib are the newly approved tyrosine kinase inhibitors for the treatment of the patients at advanced stages (1). Anti-PD-1/PD-L1 therapies may provide additional treatment options for MTC patients. More recently, in a phase 1b study, Mehnert et al. reported that the anti-PD-L1 antibody, pembrolizumab, may have an antitumor activity with an acceptable safety profile in a small percentage of advanced differentiated thyroid cancer patients (28). As with many other types of cancer, there is no clear predictive biomarker in thyroid cancers that indicates which patient will benefit more from checkpoint inhibitors. Also, PD-L1 expression is not a pure predictive biomarker for anti-PD-1/PD-L1 antibodies, but it could be useful in some cancer subtypes. In a phase one study performed by Yamamoto et al., nivolumab (anti-PD-1 antibody) found a partial response in one MTC patient. Another ongoing phase II trial (NCT03072160) explores the efficacy of pembrolizumab in recurrent and metastatic MTC (29).

Our study is the fifth study evaluating the PD-L1 positivity in MTC patients in English literature, but this study also has some limitations. This study has a retrospective design, and the number of patients is relatively small. There were only 5 PD-L1 positive tumors in our cohort, and it may not be sufficient for interpretation in the correlation analysis. Additionally, we do not have enough information about some of the patients’ distant metastasis status at the time of diagnosis. However, the median follow-up time was 54 months (min. 15 months, max. 127 months) for our cohort, and all the patients were alive during the data collection phase.

In conclusion, PD-L1 overexpression is associated with recurrence and poor prognosis in various cancers, but we did not find any significant correlation between PD-L1 expression and clinicopathologic features in our cohort. The reason may be the lack of a significant number of biologically aggressive and advanced stage (pT4) MTCs in our cohort. Further studies with larger patient numbers with advanced stage disease are still required to perform a more comprehensive analysis.

## Conflict of Interest

Authors declare no conflict of interest.
